# Implantable, 3D-Printed Alginate Scaffolds with Bismuth Sulfide Nanoparticles for the Treatment of Local Breast Cancer via Enhanced Radiotherapy

**DOI:** 10.1021/acsami.3c17024

**Published:** 2024-03-20

**Authors:** Busra Colak, Yavuz Nuri Ertas

**Affiliations:** † ERNAM—Nanotechnology Research and Application Center, Erciyes University, Kayseri 38039, Türkiye; ‡ Department of Biomedical Engineering, Erciyes University, Kayseri 38039, Türkiye; § UNAM—Institute of Materials Science and Nanotechnology, Bilkent University, Ankara 06800, Türkiye

**Keywords:** 3D printing, implantable, scaffold, bismuth sulfide, radiotherapy, breast cancer

## Abstract

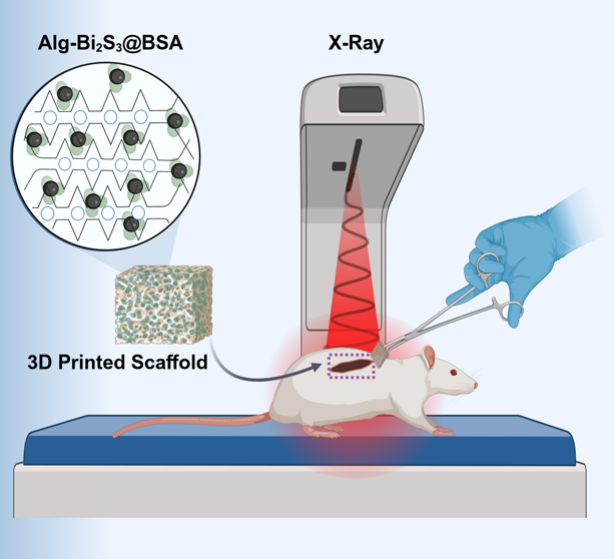

Surgical removal of tumor tissue remains the primary clinical approach for addressing breast cancer; however, complete tumor excision is challenging, and the remaining tumor cells can lead to tumor recurrence and metastasis over time, which substantially deteriorates the life quality of the patients. With the aim to improve local cancer radiotherapy, this work reports the fabrication of alginate (Alg) scaffolds containing bovine serum albumin (BSA)-coated bismuth sulfide (Bi_2_S_3_@BSA) nanoradiosensitizers using three-dimensional (3D) printing. Under single-dose X-ray irradiation in vitro, Alg-Bi_2_S_3_@BSA scaffolds significantly increase the formation of reactive oxygen species, enhance the inhibition of breast cancer cells, and suppress their colony formation capacity. In addition, scaffolds implanted under tumor tissue in murine model show high therapeutic efficacy by reducing the tumor volume growth rate under single-dose X-ray irradiation, while histological observation of main organs reveals no cytotoxicity or side effects. 3D-printed Alg-Bi_2_S_3_@BSA scaffolds produced with biocompatible and biodegradable materials may potentially lower the recurrence and metastasis rates in breast cancer patients by inhibiting residual tumor cells following postsurgery as well as exhibit anticancer properties in other solid tumors.

## 1 Introduction

Breast cancer in women has become the most prominent cancer that develops in the epithelial tissue of the breast, ranking first in both incidence and mortality.^1^ For early-stage patients, the standard care includes lumpectomy or mastectomy with lymph node sampling, followed by adjuvant radiotherapy to the tumor bed or the entire breast.^2^ Locally advanced breast cancer (LABC) patients receive multimodal therapies that involve surgery as well as adjuvant hormone, chemotherapy, and radiotherapy. When nonsurgical treatments are suitable, radiotherapy can be employed to achieve tumor regression. In such instances, radiotherapy lowers the recurrence rate of breast cancer on the ipsilateral and the associated mortality rate. Throughout the various stages of breast cancer treatment, radiotherapy is critical for enhancing local control rates and the general health of high-risk patients. Studies have demonstrated that preoperative radiotherapy does not offer significant benefits to patients, while postoperative radiotherapy has been shown to improve overall survival rates in patients with LABC.^3,4^ Thus, postoperative radiotherapy is still the primary and traditional treatment modality for LABC patients in clinical practice. Postoperative radiotherapy significantly lowers the risk of breast tumor recurrence and, to a lesser extent, the risk of distant recurrence and breast cancer death.^5^

The main therapeutic effect of radiotherapy is to induce cellular damage or apoptosis through direct or indirect interactions between cancer cell internal components and high-energy ionizing radiation (IR).^6,7^ Increasing the maximum dose accumulation in tumor tissues while at the same time reducing the damage to normal tissues is the main limitation of radiotherapy.^8^ Nanotechnology provides a variety of therapeutic strategies that can be used to overcome the radiation resistance of tumor tissue, enhance the radiation tolerance of normal tissues, increase the radiosensitivity of tumor tissues, limit the accumulation of radiation doses in the tumor volume, and balance side effects.^9,10^ Nanomaterials, which are being intensively studied and investigated to be integrated into cancer therapy, can be used as potential candidates to achieve the main optimization of radiotherapy and enhanced radiation therapy.

Nanoparticles that can increase the effect of direct and/or indirect interactions of radiotherapy as a result of their interaction with radiation are defined as radiosensitizers.^11^ In particular, radiosensitizers in the form of metal nanoparticles with high atomic number (*Z*), which are deposited in tumor tissue and have a dose-increasing effect, are preferred as an alternative therapeutic route that can overcome the mentioned limitations and improve the therapeutic window.^12^ Some high-*Z* metal nanoparticles have been proposed for breast cancer treatment, such as gadolinium (*Z* = 64), hafnium (*Z* = 72), tantalum (*Z* = 73), gold (*Z* = 79), and bismuth (*Z* = 83).^13−17^ Bismuth (Bi), which has the highest *Z* among these elements and is biocompatible and stable in forms such as sulfur and oxide, has been shown to have a greater radiosensitization effect when the particle size, concentration, and area of influence of bismuth nanoparticles are the same compared to gold and platinum nanoparticles, and they have been used in the treatment of breast cancer to increase therapeutic efficacy as radiosensitizer nanoparticles.^18−22^

Numerous strategies have been developed to target the tumor tissue to deliver therapeutic agents, which are predominantly based on passive or active targeting.^23,24^ However, studies indicate that on average, less than 1% of the injected nanoparticle dose typically accumulates in the tumor tissue, regardless of active or passive delivery approaches, and off-target accumulation is considerable.^25^ In addition, fast clearance by the reticuloendothelial system and complicated physiological barriers in vivo present formidable hurdles for drug delivery methods with limited targeting efficiency.^26^ Also, a negative verdict was recently raised regarding the enhanced permeability and retention (EPR) effect, which is the main passive targeting mechanism of nanoparticles.^27^ Therefore, new targeting strategies should be developed, depending on the application area, for dose limitation, dose repetition, and prevention of accumulation in healthy tissues.

Localized systems, which are minimally invasively implanted directly in the malignant region, can minimize excessive therapeutic agent circulation compared to systemic administration and significantly reduce the negative effects of therapeutics on normal tissues.^28^ However, secondary removal surgery may be required because the majority of current matrixes are not biodegradable. A targeting strategy to provide local enhanced radiotherapy in breast cancer treatment and to “get rid of” residual tumor cells after surgery leads to the idea that radiosensitizing nanoparticles can be implanted into the tumor site after surgical resection with a platform to carry them. The idea is to insert a scaffold containing nanoradiosensitizers into the cancerous tissue, where it can also target any remaining tumor cells. This scaffold, which may need to be implanted only once, can enhance local radiotherapy treatment while minimizing systemic toxicity. Implantable scaffolds containing nanoparticles have been produced by the three-dimensional (3D) printing technique, which allows the production of customized scaffolds for local delivery of therapeutic nanoparticles and agents to the solid tumor site.^29^ 3D bioprinting is a widely used technique to promote regeneration in fields such as tissue engineering,^30^ but in this study, it was utilized to develop a new targeting strategy to improve local delivery therapy and to enable facile and mass production of scaffolds with a better geometric fit to the patient’s anatomy. For example, Dang et al. 3D-printed and implanted the F127-SA/Cu-DOX scaffold, which provided sustained release of therapeutic agents for postoperative synergistic cancer therapy. Chemotherapy with DOX continuously released from the implanted F127-SA/Cu-DOX hydrogel scaffolds, and chemodynamic therapy with Cu(II) effectively inhibited hepatocarcinoma tumor growth.^31^ In our previous study, we fabricated alginate-based implantable scaffolds containing BSA-coated CuS nanoparticles (Alg-CuS/BSA) for local breast cancer treatment using 3D printing. Among the treatment groups under 808 nm irradiation, the synergistic effect of PTT, CDT, and laser-triggered PDT resulted in the lowest tumor volume and highest inhibition rate in the Alg-CuS/BSA + NIR group. As a result of synergistic therapy with PTT, PDT, and CDT, the tumor completely disappeared in two of the mice within 20 days, following surgical implantation. The study shows that therapeutic results achieved with synergistic therapy in cancer treatment can be much more effective.^32^ 3D printing allows for the development of more personalized hydrogels for topical treatments, including breast cancer.^33^ Nonetheless, the scientific literature on 3D-printed hydrogel-based scaffolds for the radiotherapy of breast cancer has been scarce.

**Figure 1:**
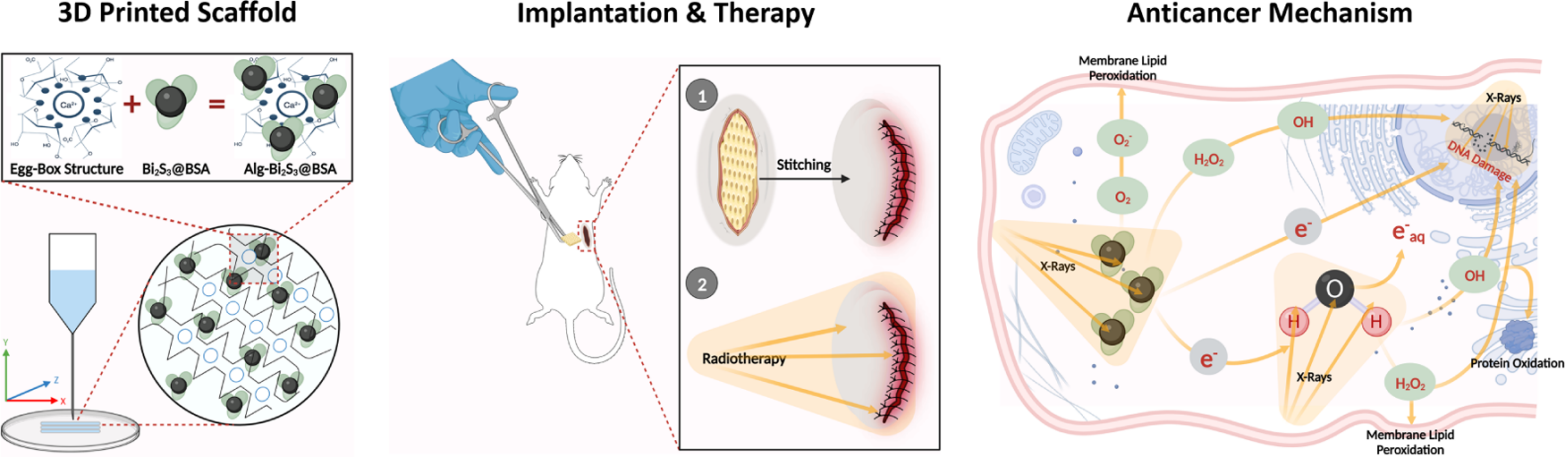
Schematic representation of the research. First, Alg-Bi_2_S_3_@BSA scaffolds were produced by using 3D printing. Second, the scaffolds were implanted beneath mouse tumor tissue and radiotherapy was applied. Third, the radiosensitization effect of implantable scaffolds under X-ray is illustrated by the given anticancer mechanism.

In this research, we fabricated high-*Z* radiosensitizer nanoparticle-loaded hydrogel-based scaffolds using 3D printing to increase the therapeutic efficacy of radiotherapy, which is widely used in the treatment of breast cancer in the clinic but has not fully demonstrated its therapeutic efficacy due to several limitations, and to provide advanced radiotherapy of breast cancer. We synthesized bismuth sulfide (Bi_2_S_3_@BSA) nanoparticles using a bovine serum albumin (BSA)-mediated biomineralization process as radiosensitizer nanoparticles. BSA acts as a sulfur precursor in Bi_2_S_3_ formation while enhancing the nanoparticle stability. The synthesized nanoparticles need to be homogeneously dispersed and carried in an implantable material in order to be locally applied to the solid tumor site. The platform that will carry the synthesized nanoparticles (Bi_2_S_3_@BSA) and enable them to be implanted into the tumor site is a biocompatible, biodegradable, easily gelable alginate (Alg) solution, which is widely used as an ink in 3D printing. Using the 3D printing technique, we produced scaffolds in the desired shape, size, and density with ink consisting of a mixture of Bi_2_S_3_@BSA and Alg solution. The scaffolds (Alg-Bi_2_S_3_@BSA) had proper mechanical strength and could be implanted into the solid tumor site. The implantable scaffolds, fabricated using a simple method and biocompatible and biodegradable materials, showed effective therapeutic efficacy in the mouse breast cancer cell line (4T1) under single-dose radiation irradiation (4 Gy) in vitro and in vivo assays and may be a potential treatment option to increase radiosensitization in breast cancer, which is one of the most common types of cancer where radiotherapy is applied and especially to prevent local recurrence after surgery. A schematic representation of the main steps of the research (scaffold fabrication, implantation, therapy, and radiosensitization effect of the scaffold) is given in Figure 1. This work, for the first time, reports a 3D-printed and implantable hydrogel scaffold containing Bi_2_S_3_@BSA nanoparticles for local tumor treatment via radiotherapy, where the radiotherapeutic potential of the scaffolds was examined in detail.

## 2 Materials and Methods

### 2.1 Materials

Bovine serum albumin (BSA), bismuth nitrate (Bi(NO_3_)_3_), and sodium hydroxide (NaOH) were obtained from Sigma, and nitric acid (HNO_3_) was obtained from Tekkim and used in the nanoparticle synthesis. Sodium alginate (Alg) was purchased from Isolab, calcium sulfate (CaSO_4_) was purchased from Sigma, and calcium chloride (CaCl_2_) was purchased from AFG Bioscience, and they were used in the fabrication of nanoparticle-loaded scaffolds. Fetal bovine serum (FBS), RPMI-1640 cell medium, penicillin/streptomycin, and trypsin EDTA were obtained from Biological Industries; MTT, dimethyl sulfoxide (DMSO), propidium iodide (PI), Calcein-AM, and crystal violet were purchased from Sigma and used in cell culture studies.

### 2.2 Preparation of BSA-Coated Bismuth Sulfide Nanoparticles (Bi_2_S_3_@BSA)

**Figure 2:**
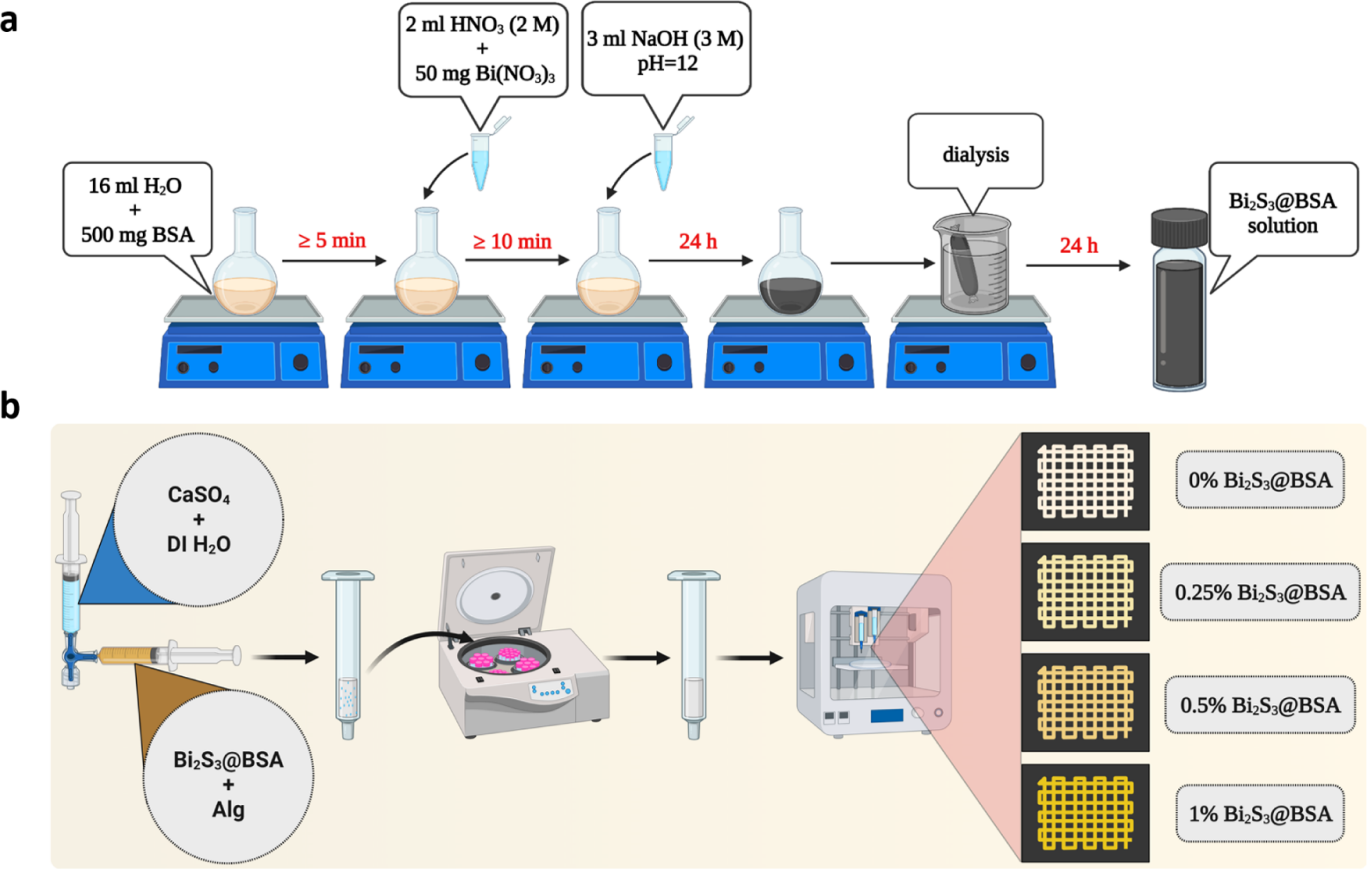
(a) Schematic representation of Bi_2_S_3_@BSA nanoparticle synthesis by the BSA-mediated biomineralization approach. (b) Schematic representation of fabrication of nanoparticle-loaded 3D-printed scaffolds.

Bi_2_S_3_@BSA nanoparticles were synthesized using a BSA-mediated biomineralization approach.^34^ 500 mg of BSA was placed on a magnetic stirrer until dissolved in 16 mL of deionized water. 50 mg of Bi(NO_3_)_3_ was dissolved in 1 mL of HNO_3_ at a 2 M concentration. Then, the prepared Bi(NO_3_)_3_ solution was slowly added to the BSA solution while being magnetically stirred. After the formation of Bi and BSA complexes, NaOH was added to the solution to biomineralize the Bi_2_S_3_ form of BSA. Stirring continued for 12 h. After the biomineralization process was complete, the colorless solution turned black after the prepared BSA stabilized Bi_2_S_3_. The final BSA-coated Bi_2_S_3_ was purified by dialysis against water for 24 h to obtain Bi_2_S_3_@BSA nanoparticle solution (Figure 2a).

### 2.3 Characterization of Bi_2_S_3_@BSA Nanoparticles

The size and shape of the obtained nanoparticles were characterized by scanning transmission electron microscopy (STEM, Zeiss Gemini SEM 500), hydrodynamic size and ζ-potential were characterized by dynamic light scattering (DLS, Malvern Instruments, NanoZS90) analysis, and the chemical state and composition of the particles were characterized by ultraviolet–visible region spectroscopy (UV–vis, PerkinElmer Lambda 25) and X-ray diffraction (XRD, Bruker Axs D8 Advance Model) analyses.

### 2.4 Preparation of Alginate Gels Loaded with Bi_2_S_3_@BSA Nanoparticles (Alg-Bi_2_S_3_@BSA)

Alginate (Alg) gels containing Bi_2_S_3_@BSA were prepared by using the cross-linking reaction according to the concentrations listed in Table 1.

**Table 1:** Amounts of Gels Containing Nanoparticles at Different Concentrations for Use in 3D Printing

alginate (Alg) (mg)	nanoparticle solution (Bi_2_S_3_@BSA) (mL)	CaSO_4_ (12.7 mg/mL) (mL)	DI H_2_O (mL)
60	0	1	2
60	0.558	1	2
60	1.116	1	2
60	2.232	1	2

Alg solution with four different nanoparticle concentrations was dissolved in separate beakers (5 mL) using Alg and deionized water (DI H_2_O), CaSO_4_, and Bi_2_S_3_@BSA nanoparticle solution to form inks for 3D printing. A three-way tap was used for gelation and homogeneous mixing. The ink mixture was taken into the printing syringe and centrifuged at the optimal values to eliminate the air bubbles. After centrifugation, the ink in the printing syringe was ready to be used for 3D printing.

### 2.5 Fabrication of 3D-Printed Alg-Bi_2_S_3_@BSA Scaffolds

Axo A3 Bioprinting device (Axolotl, Türkiye) was used for 3D printing of alginate scaffolds containing several concentrations of nanoparticles. After centrifugation, a 0.5 mm diameter needle (25 G) was attached to the tip of the printing syringe containing the ink mixture. The scaffold dimensions required for each experiment were updated with Repetier Host software, and Alg scaffolds with different nanoparticle concentrations were produced by extruding them onto a Petri dish along *X*–*Y*–*Z* routes at optimal pressure and temperature values by using pneumatic printing (Figure 2b). After printing, the scaffolds were immersed in a CaCl_2_ solution to undergo a secondary cross-linking process.

### 2.6 Characterization of 3D-Printed Alg-Bi_2_S_3_@BSA Scaffolds

The morphological assessment of the 3D-printed scaffolds was carried out using a scanning electron microscope (SEM, Zeiss Leo 440), and elemental analysis using energy-dispersive X-ray spectroscopy (EDX or EDS). In addition, the compressive moduli of the 3D scaffolds with a width of 15 mm and a thickness of 7 mm were measured at room temperature by using a dynamic mechanical analyzer (TA DMA Q800), in which a uniaxial compressive force was applied with a displacement rate of 2 mm/min.

### 2.7 In Vitro Degradation of Scaffolds

The 3D-printed scaffolds were additionally tracked over time to ascertain their physical integrity and stability. Printed grid scaffolds were placed in a well plate and submerged in 1000 μL of PBS. The scaffolds were imaged and observed on different days for 16 days.

### 2.8 In Vitro Assays

#### 2.8.1 Radiosensitization Effect of Alg-Bi_2_S_3_@BSA Scaffolds on Cancer Cell Viability

Mouse breast cancer cells (4T1) at a density of 5 × 10^3^ per well were cultured in RPMI-1640 medium (10% PBS, 1% penicillin/streptomycin) at 37 °C and 5% CO_2_ for 24 h. Then, scaffolds were placed in 96-well plates according to the determined groups. The experimental groups are as follows: Control (no treatment), X-ray, only alginate (Alg) scaffold, Alg-Bi_2_S_3_@BSA (0.25%) + X-ray, Alg-Bi_2_S_3_@BSA (0.5%) + X-ray, Alg-Bi_2_S_3_@BSA (1%) + X-ray. 5 h after the scaffolds were placed, the groups to be treated with X-ray irradiation were exposed to X-ray irradiation (4 Gy, 6 MV). The MTT assay was used to determine the killing effect of each experimental group on cancer cells. 20 μL of MTT (5 mg/mL) was added to each well 24 h after treatment. Then, after an additional 4 h of incubation, 100 μL of DMSO was added to the cell medium. To determine the percentage of cell viability, the absorbance of formazan was recorded at 570 nm with a microplate reader.

#### 2.8.2 Colony Formation Assay

For the colony formation assay, 4T1 cells were cultured in plates at a density of 300 cells per well and incubated in an incubator at 37 °C for 48 h. The cells were then treated with different treatment groups: Control, X-ray, only Alg, Alg-Bi_2_S_3_@BSA (1%), Alg-Bi_2_S_3_@BSA (1%) + X-ray. After 4 h of incubation, the culture media in all wells were removed and replaced with fresh culture media after washing with PBS. Groups with irradiation in their treatment plan were irradiated with X-ray and cancer cells were incubated for 8 days to obtain colony information. After discarding the culture medium and washing with PBS, cells were removed before staining with 0.5% crystal violet in a methanol/acetic acid mixture (3:1). After 15 min of incubation, the plates were immersed in water to remove the crystal violet color and allowed to dry overnight at room temperature. Colonies were analyzed based on the blue-violet color ratio through graphical and visual interpretation. The formulas for calculating the survival fraction are given below
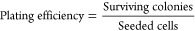

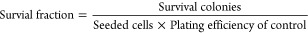


#### 2.8.3 Live and Dead Cell Staining Assay

4T1 cells were cultured in a 96-well plate and incubated in an incubator for 24 h. After incubation, 4T1 breast cancer cells treated with scaffolds for 5 h were treated with the following experimental groups: Control, X-ray, only Alg, Alg + X-ray, Alg-Bi_2_S_3_@BSA (1%), and Alg-Bi_2_S_3_@BSA (1%) + X-ray. Calcein-AM (100 μL, 3 μM) and PI (100 μL, 4 μM) solutions were then added to the wells to determine live and dead cells. Fluorescence microscope (Leica DM IL LED Fluo, 510081) was utilized to visualize the staining results.

#### 2.8.4 Evaluation of Intracellular ROS Generation in Cancer Cells

4T1 cells seeded in 96-well plates were incubated in an incubator for 24 h at the values given in previous assays. Then, the treatment groups were applied to the cancer cells: Control, X-ray, only Alg, Alg + X-ray, Alg-Bi_2_S_3_@BSA, and Alg-Bi_2_S_3_@BSA + X-ray. After the incubation period, DCFH-DA was added to the 4T1 cells treated with the scaffolds for 5 h, and the cells were incubated for another 1 h. Fluorescence microscope (Leica DM IL LED Fluo, 510081) was utilized to visualize the results.

### 2.9 In Vivo Anticancer Studies

#### 2.9.1 Generating and Treating Breast Tumor Tissue in a Mouse Model

To evaluate the radiosensitizer efficacy of scaffolds in an animal model, 200 μL of cell suspension (containing 1 × 10^6^ 4T1 cells) were injected subcutaneously to generate breast tumor tissue on the right shoulder of BALB/C mice. The therapy was initiated when the tumor volume reached approximately 200 mm^3^ (10–14 days) after inoculation. Tumor volume was determined by using the following formula
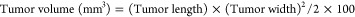


After tumor tissue formation, tumor-bearing mice were randomly divided into four groups (*n* = 5 per group). The therapy groups were as follows: Control, X-ray, Alg-Bi_2_S_3_@BSA (1%), Alg-Bi_2_S_3_@BSA (1%) + X-ray. Tumor-bearing mice were anesthetized by intraperitoneal injections of ketamine and xylazine. The scaffolds were then implanted into the tumor site. Targeted mice were exposed to a single dose of X-ray (4 Gy, 6 MV) 24 h after implantation. The tumor size and weight of the mice during the treatment period were observed and documented on different days for 16 days.

#### 2.9.2 Histopathology Analysis

The major organs of the mice were examined using the histopathology analysis of hematoxylin and eosin (H&E) staining. After the mice were sacrificed, the main organs (heart, liver, spleen, and kidney) were removed and placed in a 4% paraformaldehyde solution. Then, 5 mm sections were taken from the organs, and stained with H&E.

### 2.10 Statistical Analysis

All data were expressed as mean ± SD unless otherwise claimed, and GraphPad Prism software was utilized for performing statistical analyses.

## 3 Results and Discussion

### 3.1 Synthesis and Characterization of Bi_2_S_3_@BSA Nanoparticles

Bi_2_S_3_@BSA nanoparticles were synthesized as nanoradiosensitizers via a BSA-mediated biomineralization approach. The synthesis method is characterized by two basic steps.

(i) Complexation of BSA with Bi^3+^ ions occurred under acidic conditions, and (ii) pH-dependent formation of Bi_2_S_3_ nanoparticles under basic conditions was achieved by adjusting the reaction pH to 12.

Functional groups (e.g., –SH, –NH_2_, −COOH) in BSA form the BSA-Bi^3+^ complex with Bi^3+^ ions in Bi(NO_3_)_3_ under acidic conditions. BSA denatures under strong basic conditions to release a large number of cysteines. The released cysteines are the source of sulfur needed to form metal sulfide nanoparticles. Therefore, BSA acts as a stabilizer and sulfur source for the formation of Bi_2_S_3_ nanoparticles.^34^

**Figure 3:**
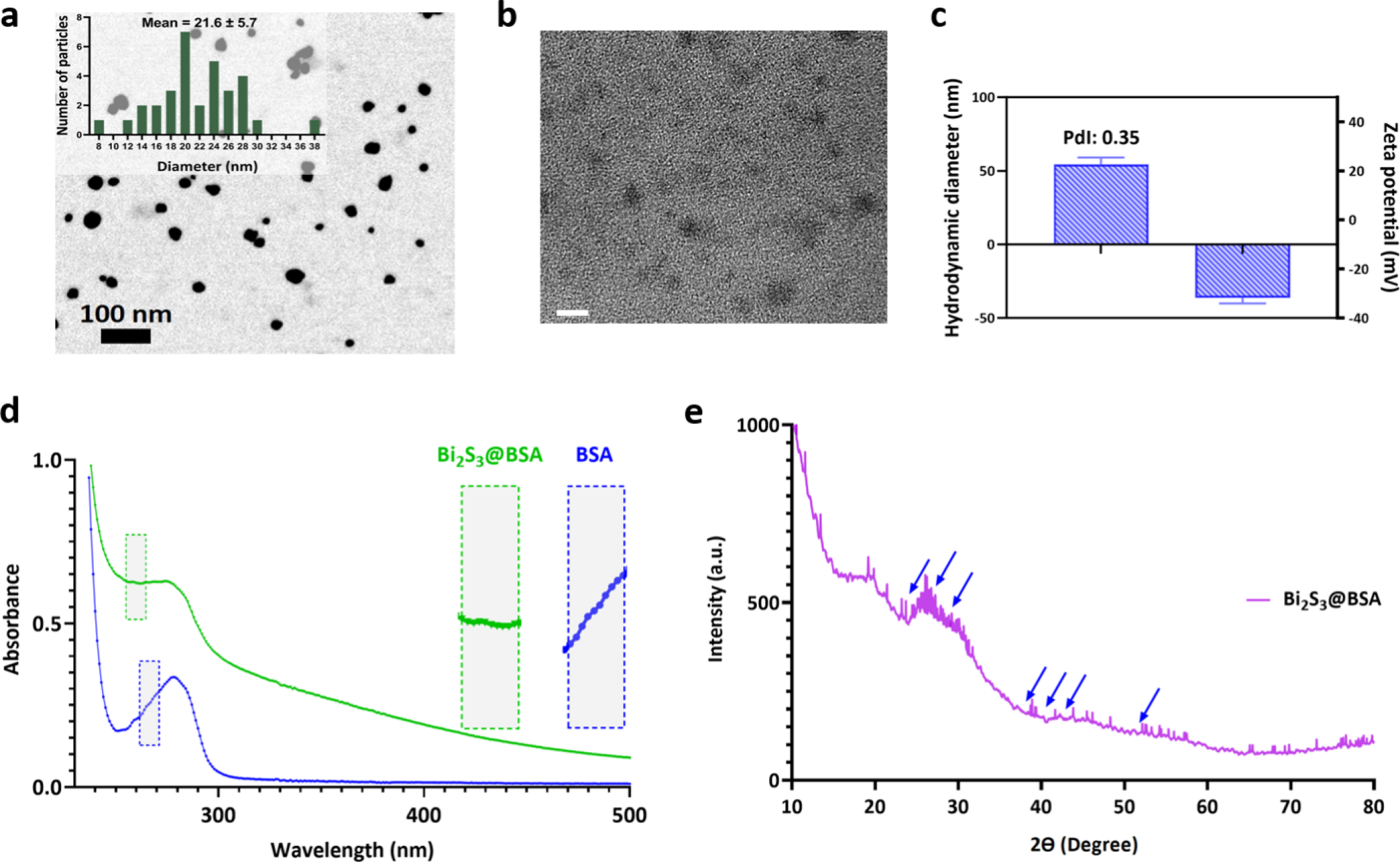
Characterization of nanoparticles (Bi_2_S_3_@BSA). (a) STEM image of Bi_2_S_3_@BSA nanoparticles and the corresponding size distribution histogram of the STEM image. (b) TEM image of Bi_2_S_3_@BSA nanoparticles (scale bar = 20 nm). (c) Graphical representation of hydrodynamic size diameter and zeta potential. (d) UV–vis spectrum of BSA and Bi_2_S_3_@BSA. (e) XRD pattern of the Bi_2_S_3_@BSA nanoparticles.

STEM and TEM were used to determine the size and morphology of the synthesized nanoparticles. The nanoparticles were spherical, monodisperse, and had a uniform size distribution with a mean diameter of 21.6 (Figure 3a,3b). The hydrodynamic size distribution and ζ-potential of Bi_2_S_3_@BSA were determined via DLS analysis. Among the graphical data obtained by DLS analysis, especially density-dependent size distribution plots are reliable, as they show even small amounts of aggregation. The average size distribution of nanoparticles with respect to density was 54.47 nm, and the polydispersity index (PDI) indicating size heterogeneity was 0.351 (Figures 3c and S1). The ζ-potential value, which indicates the surface charge of the nanoparticles and represents colloidal stability, was −36.3 mV (Figures 3c and S1). Negative surface charge results from the existence of BSA coating.^35^ In addition, the size of Bi_2_S_3_@BSA nanoparticles measured by TEM was smaller than those observed by DLS. Due to the presence of BSA and the swelling of the nanoparticles in an aqueous solution, it may be assumed that the hydrodynamic diameter of the nanoparticles increases. The fact that the size obtained by DLS measurement is different from that of TEM can be attributed to the hydration of the particles in the DLS method.^36^

UV–vis and XRD analyses were performed to establish the chemical structure and composition of the nanoparticles. The UV–vis spectra peaks of BSA and Bi_2_S_3_@BSA nanoparticles were around 266 and 264 nm, respectively. Additionally, noticeable absorption was observed in the spectrum of Bi_2_S_3_@BSA nanoparticles at 265 nm, indicating that the nanoparticles were coated with BSA (Figure 3d).^37^ According to the results of the XRD analysis, the characteristic diffraction peaks of the nanoparticles are shown with blue arrows, and Bi_2_S_3_@BSA nanoparticles were verified by the JCPDS database (JCPDS 17-0320).^19^ The broad and diffuse peaks in the XRD pattern indicate that the structure is amorphous, while the BSA coating was verified by the emergence of a broad and moderately intense peak in the range of 20–29°, which corresponds to the XRD pattern of the BSA (Figure 3e).^38^ Sulfur-based proteins (such as BSA) provide oriented metallic nanoparticle formation, a specific nucleation site, and uniform-sized nanoparticles and allow the nanoparticles to remain stable against environmental conditions such as temperature, pH, and concentration.^39^ In addition, BSA-coated nanoparticles can be preferentially uptaken by cancer cells.^40^

### 3.2 Fabrication and Characterization of 3D-Printed Alg-Bi_2_S_3_@BSA Scaffolds

**Figure 4:**
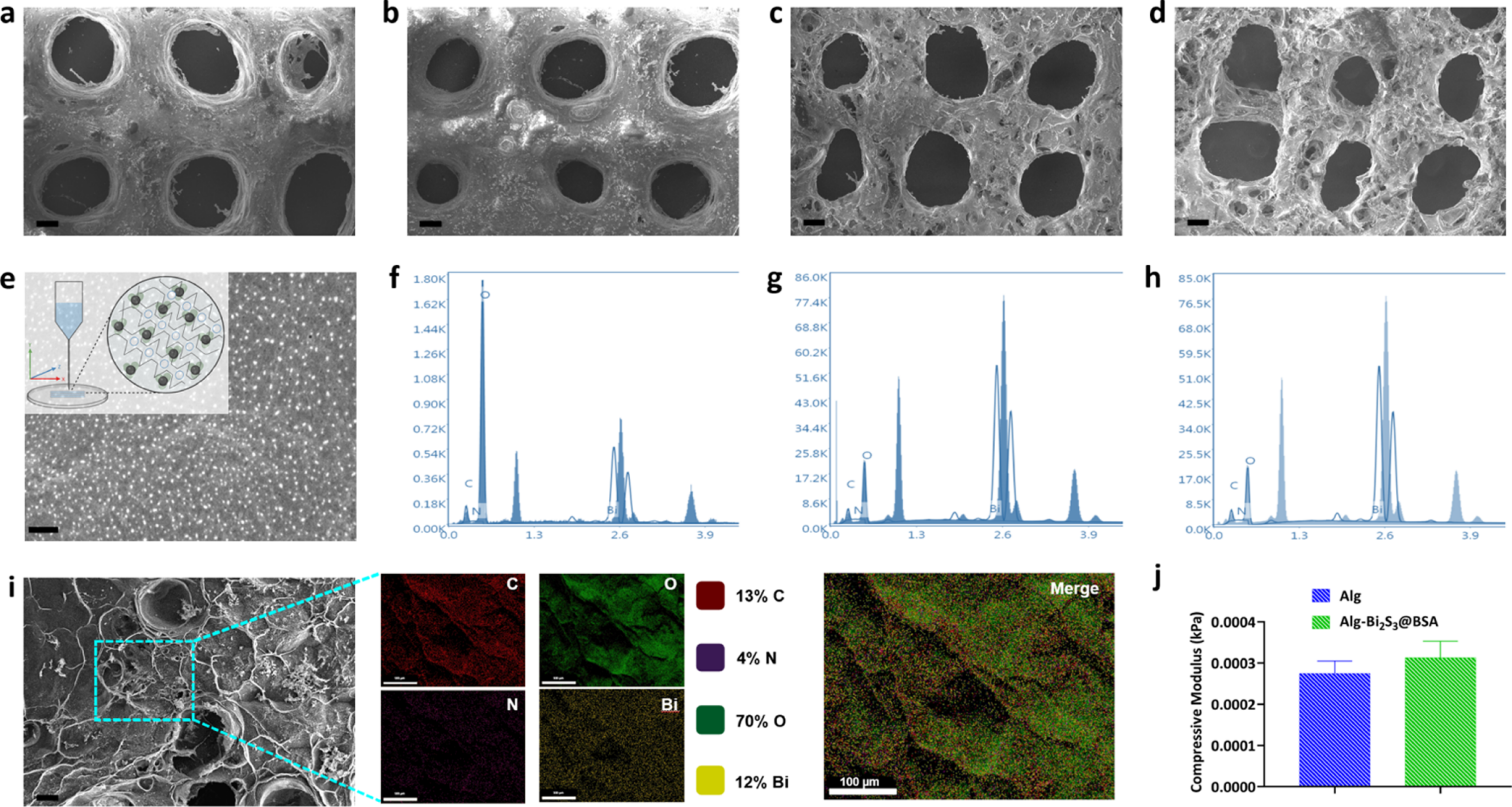
Characterization of the Alg-Bi_2_S_3_@BSA scaffolds. FESEM images of (a) Alg scaffold (Scale bar = 100 μm), (b) Alg-Bi_2_S_3_@BSA (0.25%) (scale bar = 200 μm), (c) Alg-Bi_2_S_3_@BSA (0.5%) (scale bar = 100 μm), and (d) Alg-Bi_2_S_3_@BSA (1%) (scale bar = 200 μm). (e) SEM image of Alg-Bi_2_S_3_@BSA (1%) scaffold (white dots are Bi_2_S_3_@BSA nanoparticles, scale bar = 100 nm). EDS spectrum of (f) Alg-Bi_2_S_3_@BSA (0.25%), (g) Alg-Bi_2_S_3_@BSA (0.5%), and (h) Alg-Bi_2_S_3_@BSA (1%). (i) EDS elemental mapping of the Alg-Bi_2_S_3_@BSA (1%) scaffold (scale bar = 100 μm). (j) Compressive moduli of scaffolds (Alg and Alg-Bi_2_S_3_@BSA (1%), respectively).

Alginate is frequently preferred as the main scaffold material for extrusion-based 3D printing due to its controllable rheological properties, biocompatibility, biodegradability, hydrophilicity, microporosity, and physical cross-linking.^41^ CaCl_2_ is often preferred as a physical binder for the pre-cross-linking of alginate, but CaCl_2_ has a high solubility in water and leads to the uncontrolled release of Ca^2+^ ions, causing heterogeneous cross-linking and forming an unstable external gelation.^42^ Cross-linkers with sulfate salts such as CaSO_4_ have lower solubility and uniform cross-linking, and the high Young’s modulus and equilibrium modulus lead to better structural integrity after printing.^43^ Therefore, gelation via CaSO_4_ was chosen. Then, the alginate-based scaffolds containing Bi_2_S_3_@BSA nanoparticles (Alg-Bi_2_S_3_@BSA) were fabricated by using a 3D bioprinting device (Figure S2) with the parameters given in Table 1 and Table S1. The surface morphology of the produced Alg-Bi_2_S_3_@BSA scaffolds were examined via FESEM. Only Alg and other scaffolds containing different concentrations of nanoparticles had microscale pores with a diameter of about 400 μm (Figure 4a–d). Increasing nanoparticle concentrations changed the surface morphology, promoting the surface roughness and causing the formation of micro- and nanosized pores within the structure. The negligible degradation of macrostructured pores and increased nanoporosity of the scaffolds were attributed to Bi_2_S_3_@BSA nanoparticles integrated into the alginate hydrogel. The alginate scaffold, which will act as a carrier platform for the synthesized nanoparticles, was produced by the gelation of CaSO_4_ and sodium alginate (egg box structure). In the SEM image of the scaffold (Alg-Bi_2_S_3_@BSA (1%)), the nanoparticles were homogeneously distributed on the surface (Figure 4e). 3D printing allowed the nanoparticles to spread homogeneously in the printed alginate gel. Bi_2_S_3_@BSA nanoparticles, which were homogeneous and monodisperse on the scaffold surface, are expected to exhibit a homogeneous radiotherapeutic effect in tumor tissue under X-ray irradiation upon implantation.

EDS analysis can be utilized to identify the elemental composition of specific points or to map out the lateral distribution of elements from the imaged region. EDS analysis of Alg-Bi_2_S_3_@BSA (0.25%), Alg-Bi_2_S_3_@BSA (0.5%), and Alg-Bi_2_S_3_@BSA (1%) scaffolds confirmed the main elements (C, O, N, Bi) in the structure (Figure 4f–4h, respectively). EDS analysis of alginate scaffolds containing different concentrations of nanoparticles confirmed the presence of Bi_2_S_3_@BSA nanoparticles in the scaffold, as it revealed the major component (bismuth and Bi) of the synthesized nanoparticle. With increasing nanoparticle concentration, the amount of the main element bismuth (Bi), which causes the structure to show radiosensitizer properties, increased linearly (Figure S3). The elemental mapping of the scaffold with the highest nanoparticle concentration (Alg-Bi_2_S_3_@BSA (1%)) and the main image are given (Figure 4i).

**Figure 5:**
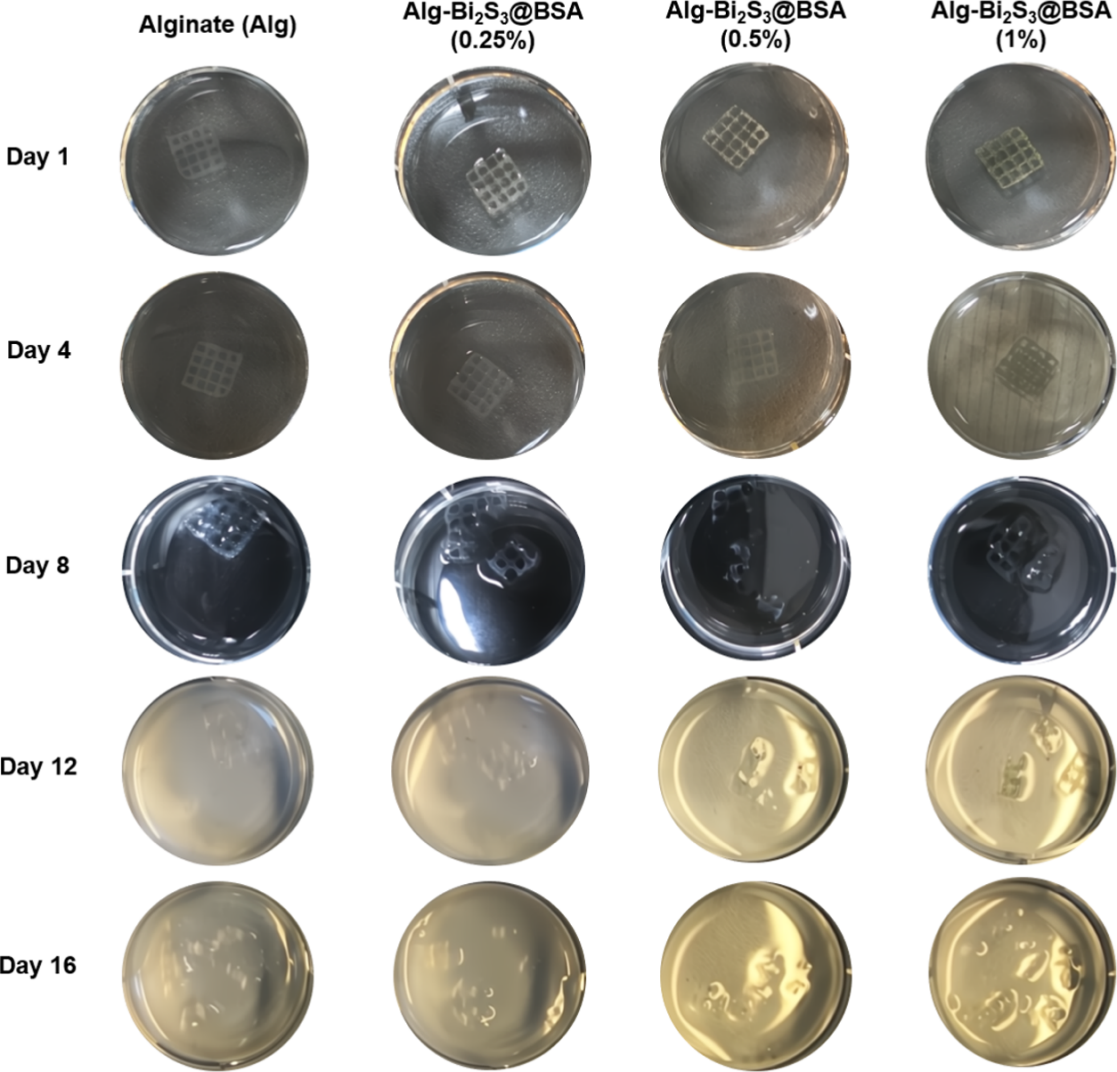
Images of scaffolds in PBS tracked over time demonstrating their degradation.

The 3D scaffolds must have implantable mechanical strength and integrity. Pure Alg scaffolds and Alg scaffolds with the highest nanoparticle concentration (Alg-Bi_2_S_3_@BSA (1%)) were prepared for mechanical testing by using 3D printing. To measure the compression moduli of the scaffolds, a compression force was applied to the scaffolds with a displacement rate of 2 mm/min using a uniaxial compression force mechanical analyzer. According to the data obtained from the slope of the first linear region of the compression modulus curve resulting from the applied compressive force, the Alg-Bi_2_S_3_@BSA (1%) scaffold showed higher mechanical strength than the pure Alg scaffold (0.000313 and 0.000275 kPa, respectively, Figure 4j). However, the moduli of the scaffolds produced were considerably lower than that of human breast tissues (3.25 ± 0.91 kPa).^44^ A proper degradation process is essential to maintaining the desired shape of scaffolds for a sufficient period of time to successfully fulfill their purpose when implanted. 3D-printed scaffolds were visually tracked in PBS at intervals of 16 days (Figure 5, Table S2). While the scaffolds began to partially degrade within 1 week, the structural integrity of the scaffolds containing nanoparticles deteriorated faster. At the end of the 16th day, all scaffolds completely lost their structural integrity.

Many studies in the literature use the intravenous (IV) route for nanoparticle delivery to solid tumors with either a passive or active targeting strategy by altering the physicochemical properties of the nanoparticles. However, the low efficiency of targeting tumor tissue via IV delivery and the controversial published findings require the treatment regimen to include various strategies aimed at overcoming different biological barriers. As an alternative to IV delivery, locally targeting tumor tissue with hydrogels may increase bioavailability, enable sustained release by time-dependent hydrogel degradation, allow high loading of therapeutic agents, and minimize exposure to normal tissues.^45^ For example, Dang et al. fabricated chemotherapeutic drug (DOX)-loaded PCL scaffolds with macropores of 300–500 μm and showed high loading efficiencies of up to 90%. They found that implantation of DOX-containing scaffolds resulted in lower cardio-cytotoxicity, decreased local cancer recurrence, and a lower progression of metastases in the lungs, liver, and spleen compared to a one-time IV injection.^46^ In short, in contrast to commonly used targeting strategies, a controllable and localized treatment can maximize the curative effect while minimizing side effects. Therefore, in this study, we aimed to locally target nanoradiosensitizers with 3D-printed hydrogel scaffolds to increase the antitumor efficacy of radiotherapy.

### 3.3 In Vitro Anticancer Assays

#### 3.3.1 Radiosensitization Effects of Alg-Bi_2_S_3_@BSA Scaffolds on Breast Cancer Cell Viability

**Figure 6:**
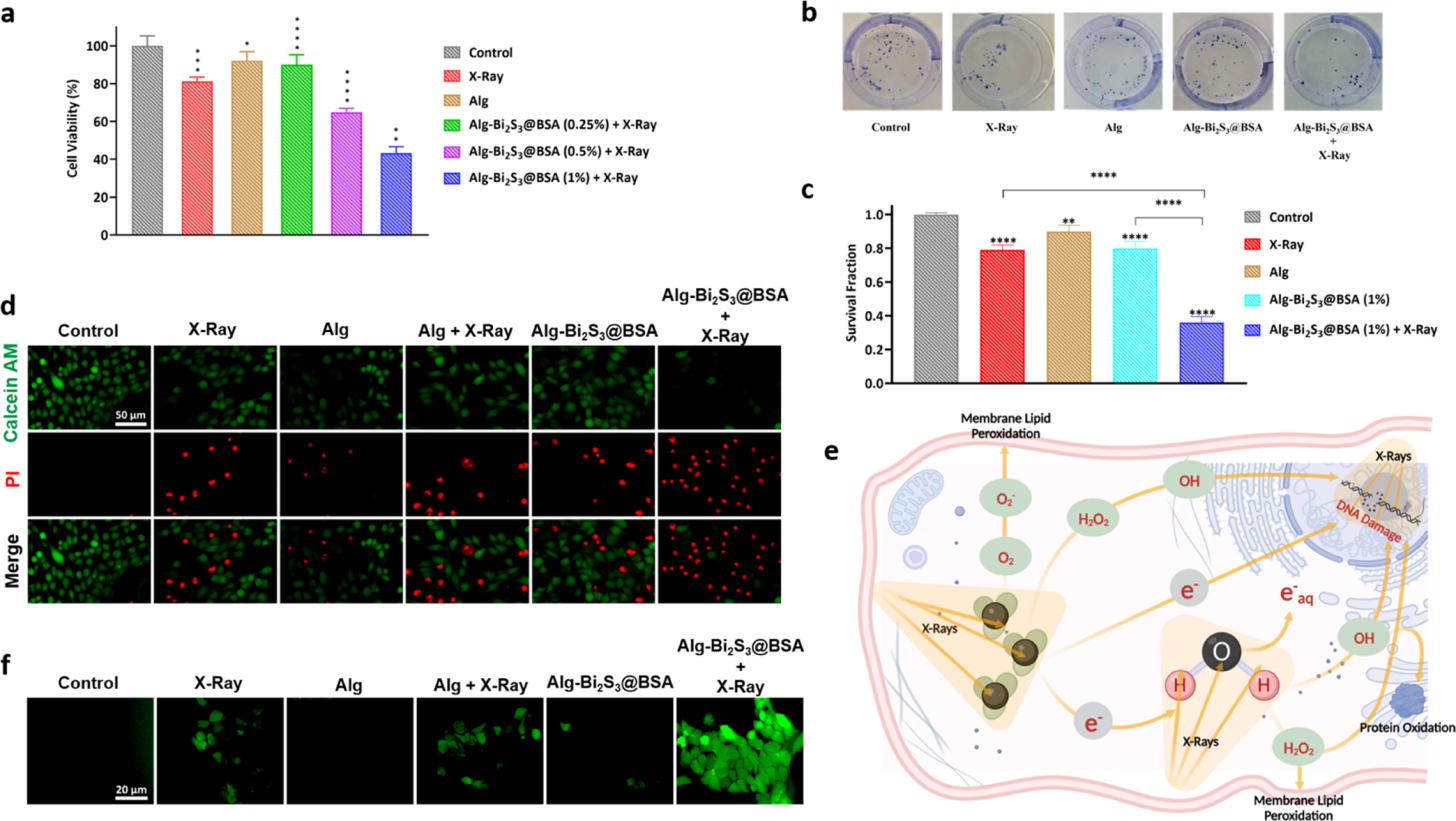
In vitro assays. (a) Cell viability test on 4T1 cells incubated with various treatments in the presence and absence of X-ray irradiation. (b) Representative images of colony formation of cells after treatment with different groups. (c) Inhibitory effects of X-ray in the presence of various treatments on the colony formation of 4T1 cells related to survival fraction. (d) Fluorescence images of 4T1 cells after various treatments with Calcein-AM/PI cell staining. (e) Radiosensitization mechanism of the interaction between incident X-ray and high-*Z* nanoparticles. (f) Intracellular ROS production after various treatments in the presence and absence of X-ray irradiation. Data are presented as mean ± SD; *p < 0.05, **p < 0.01, ***p < 0.001 and ****p < 0.0001 different compared with the control group.

MTT assay was performed to reveal the in vitro radiosensitization effects of 3D-printed Alg-Bi_2_S_3_@BSA scaffolds on the mouse breast cancer cell line (4T1) and determine the effective concentration range. Compared to the control group without any treatment, the cancer cell viability rates of the other treatment groups on 4T1 cancer cells were 81.2, 92.1, 90, 65, and 43.3%, respectively (Figure 6a). The X-ray-treated group showed very low anticancer activity (81.2%), indicating that radiotherapy treatment alone was not effective. The treatment group with the lowest nanoparticle concentration (Alg-Bi_2_S_3_@BSA (0.25%)) had less effect on cell viability (90%) than the group exposed to X-ray irradiation alone and showed a similar viability rate as the group treated with Alg only (92.1%) due to its low content of radiosensitizer nanoparticles. The scaffolds with nanoparticles could inhibit the growth of 4T1 cells in a concentration-dependent manner, and the cell viability remarkably decreased after treatment with Alg-Bi_2_S_3_@BSA + X-ray. With increasing nanoparticle concentrations, the viability of 4T1 cells decreased and anticancer activity increased under X-ray irradiation. The group treated with the scaffold with the highest nanoparticle concentration (Alg-Bi_2_S_3_@BSA (1%)) and exposed to X-ray was the most effective therapeutic group, showing viability below 50%. The decreasing viability rate of breast cancer cells with increasing radiosensitizer nanoparticle concentration under X-ray irradiation reveals that the scaffolds exhibit radiosensitization property to increase the therapeutic efficacy of radiotherapy.

#### 3.3.2 Colony-Forming Efficiency Assay

The colony formation or colony-forming efficiency assay (CFE) is based on the capacity of single cells to undergo “unlimited” division and to grow into colonies. The CFE assay can measure both cell survival and cell death (by comparing formed colonies having a blue-violet color mixture, Figure 6b, and by comparing the number of colonies in test sample plates with the number of colonies in control plates, Figure 6c). To evaluate proliferative damage and colony formation, a colony assay on the 4T1 breast cancer cell line was conducted. According to the results, X-ray-treated groups were effective in reducing colony formation compared to the control group. Without X-ray irradiation, the scaffold by itself could not provide an effective reduction in colony formation. In the therapy group treated only with X-ray, radiotherapy alone was insufficient to limit the colony abilities of cancer cells. The ability of cancer cells to form colonies decreased, especially when exposed to Alg-Bi_2_S_3_@BSA (1%) + X-ray treatment (Figure 6c). As a result, X-ray irradiation of scaffolds containing radiosensitizers increased radiotherapeutic efficacy and limited cancer cell repopulation.

#### 3.3.3 Live and Dead Cell Staining Assay

Live and dead cell staining tests rely on a detection approach in which one dye stains live cells, and another stains dead cells selectively. In the live and dead cell staining assay, which is based on the mechanism of checking cell membrane integrity and robustness, Calcein-AM interacts with the cytoplasm of living cells and emits green fluorescence, while propidium iodide (PI) interacts with the nuclei of dead cells and emits red fluorescence. The death of 4T1 breast cancer cells induced by nanoparticle-loaded scaffolds upon X-ray irradiation was evaluated using the Calcein-AM/PI staining method. 4T1 cancer cells treated with the designated treatments were stained with Calcein-AM and PI and visualized using a fluorescence microscope. Calcein-AM and PI staining of each group are shown separately and merged (Figure 6d). No cell death was observed in the control group. Cell viability was higher than cell death in X-ray, Alg, Alg + X-ray, and Alg-Bi_2_S_3_@BSA groups. The therapy group with the lowest cell viability and the highest anticancer activity on 4T1 cancer cells was the scaffold containing the highest amount of nanoradiosensitizers which was exposed to X-ray irradiation (Alg-Bi_2_S_3_@BSA + X-ray), where strong red fluorescence (dead cells) and very weak green fluorescence (live cells) was detected.

#### 3.3.4 Evaluation of Intracellular ROS Generation

Through the radiolysis of water molecules, high-*Z* nanoparticles produce ROS byproducts, which drive cell death by a variety of mechanisms, such as apoptosis, necrosis, mitotic cell death, autophagy, and permanent cell cycle arrest, and lead to several types of defects, such as DNA base damage and protein modification (e.g., cross-linking, oxidation). Incident X-ray also damages DNA (e.g., single-strand breaks and double-stranded breaks) through direct or indirect effects. With X-ray irradiation, the increased formation of ROS and secondary electrons from the high-*Z* nanoparticles result in cytotoxic damage to cancer cells (Figure 6e).^48^ Therefore, the aim is to increase DNA damage through an indirect effect by increasing intracellular ROS production using radiosensitizer nanoparticles. To assess ROS production, DCFH-DA, a ROS probe that measures hydroxyl, peroxyl, and other ROS activities in the cell, was utilized. DCFH-DA is hydrolyzed and deacetylated to DCFH by intracellular esterases. DCFH is then oxidized to highly fluorescent DCF in the presence of ROS, emitting green fluorescence (Figure S4). ROS production was not observed in the control group or in the group treated with the Alg-Bi_2_S_3_@BSA scaffold (no green fluorescence). A high rate of ROS production was observed, especially in the X-ray-treated groups (Figure 6f). Under X-ray irradiation, the nanoradiosensitizer-loaded scaffold (Alg-Bi_2_S_3_@BSA + X-ray) had the highest ROS production, damaging the metabolic activities of cancer cells through an indirect mechanism of action by increasing ROS production under X-ray.

### 3.4 In Vivo Anticancer Studies

**Figure 7:**
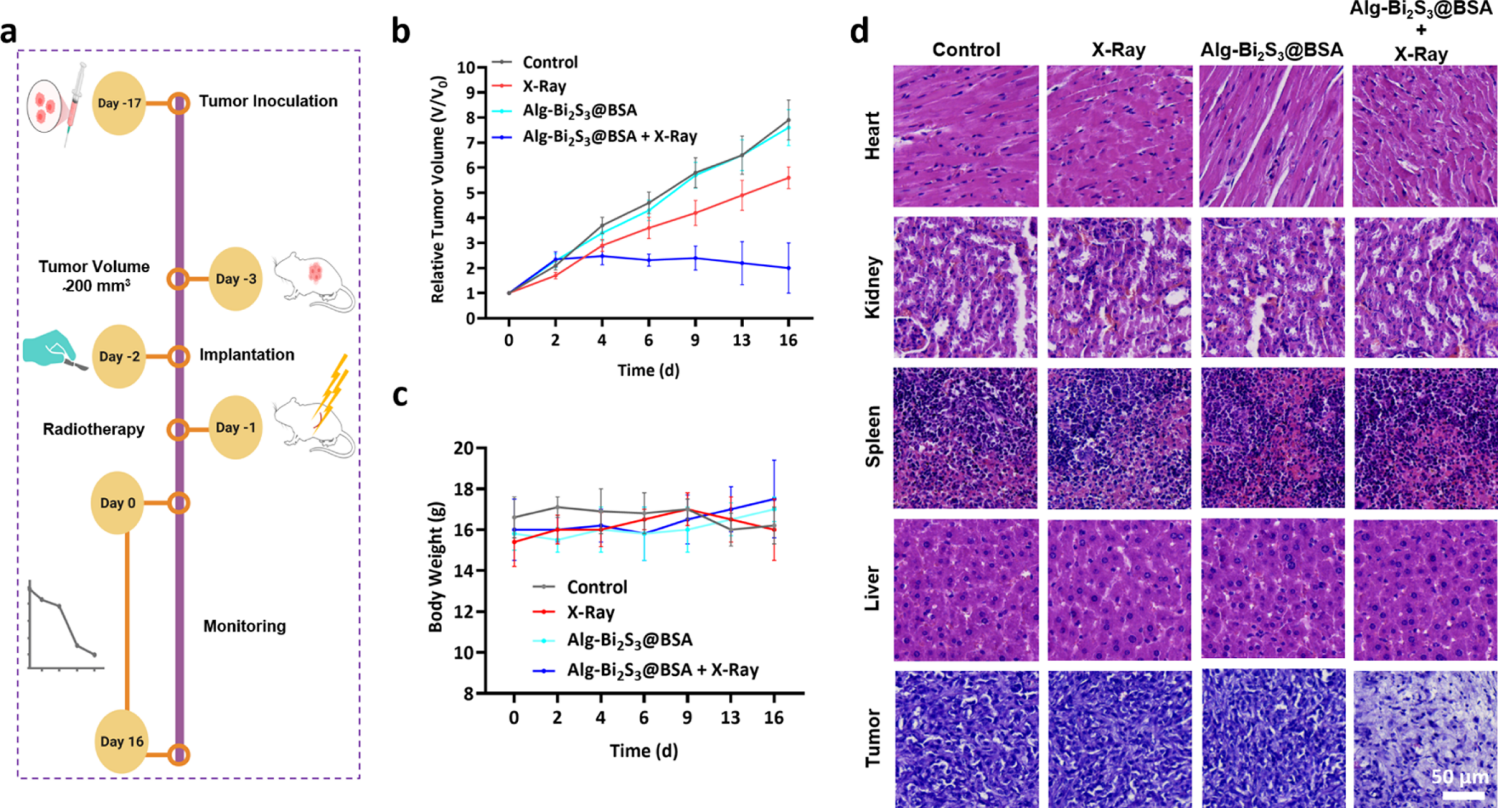
In vivo anticancer studies. (a) Schematic representation of in vivo studies. (b) Relative tumor volumes following different treatments with or without X-ray irradiation. (c) Body weight of mice with different treatments. (d) H&E staining of the main organs and tumor in the presence and absence of X-ray.

Surgical resection cannot entirely eradicate all of the tumor cells, and large breast defects always remain, which are difficult to self-heal.^49^ The local cancer treatment potential of implantable 3D-printed alginate scaffolds loaded with nanoradiosensitizers was investigated in mice bearing breast cancer tumor tissue. After the tumor reached a certain volume (∼200 mm^3^) in mice, the scaffolds with the final formulation (Alg-Bi_2_S_3_@BSA (1%)) were surgically implanted beneath the tumor, and the incision was closed by suturing (Supporting Information Video). 24 h after implantation, a single dose of conventional X-ray irradiation (4 Gy, 6 MV) was administered to the treatment groups that required X-ray exposure. After radiotherapy, the tumor volumes and body weights of mice were monitored and recorded at regular intervals for 16 days (Figure 7a).

The rate of volume enhancement of control, X-ray, and Alg-Bi_2_S_3_@BSA-treated tumor tissues increased throughout the treatment period. Radiotherapy alone failed to reduce or keep the tumor volume growth rate constant. The Alg-Bi_2_S_3_@BSA + X-ray group was the only treatment group that reduced the tumor volume increase rate (Figure 7b). Under single-dose X-ray irradiation, the scaffold containing nanoparticles exhibited tumor inhibition. The body weight of the mice was also recorded during the treatment period to physically evaluate the biocompatibility of the implantable scaffolds. While the body weight decreased in the control and X-ray-only groups, the body weight increased in the treatment groups with scaffolds (Figure 7c). Therefore, the implantable scaffolds did not cause any visible toxic effects. These results obtained under single-dose X-ray confirm that scaffolds containing nanoparticles have improved radiotherapeutic efficacy in vivo.

The major organs (heart, kidney, spleen, and liver) in mice were pathologically monitored for any risk threatening biocompatibility. Among the treatment groups, the histopathology results of the tumor tissue in the Alg-Bi_2_S_3_@BSA + X-ray treated group showed that the necrotic and more shaded tissue appearance was replaced by a significant decrease in tumor cell nuclei size and cytoplasmic formations compared to the control group (Figure 7d). The absence of visible tissue damage in the major organs proves that implantable nanoparticle-loaded scaffolds are biocompatible for use in local breast cancer treatment in vivo.

Because alginate cannot be degraded in the digestive system of mammals, it is removed from the body by hydrolytic, instead of enzymatic degradation.^50^ The alginate scaffolds produced at different concentrations remain stable in PBS for the first 3 days and after this period, the degradation slows down with increasing polymer concentration.^51^ Elsewhere, 72 h of incubation of Bi_2_S_3_@BSA nanoparticles in PBS revealed no Bi^3+^ release. The deposition of these nanoparticles in major organs and tumor tissue was also examined, and it was reported that they accumulated mainly in reticuloendothelial organs such as liver and spleen.^34^ Bi_2_S_3_@BSA-loaded alginate scaffolds produced using biocompatible materials at a tolerable level will be excreted from the body with minimal toxicity after treatment.

The in vitro and in vivo results show that the produced scaffolds inhibit breast cancer growth under X-ray irradiation. These scaffolds hold promise for enhanced radiotherapy of solid tumors, and by modifying (e.g., chemotherapeutics, immunotherapeutics) or enhancing (e.g., drug-loaded nanoparticles) therapeutic agents, they could be used as a primary or complementary procedure to the desired treatment modality.^33^ Biodegradable implants can be fabricated according to the patient’s specific anatomy by 3D printing and do not require a secondary surgical procedure for removal, therefore, they have great potential for postoperative cancer treatment due to increased drug dosage and reduced systemic toxicity in the disease area and support the concept of personalized medicine with a therapeutic dose appropriate to the patient.^52^ Because such scaffolds also have the potential to provide tissue repair after tumor removal, they will be at the forefront of future biomedical research.

## 4 Conclusions

In summary, we fabricated Bi_2_S_3_@BSA nanoparticle-loaded alginate scaffolds utilizing 3D printing to provide enhanced local radiotherapy treatment for local breast cancer. Bi_2_S_3_@BSA radiosensitizer nanoparticles were synthesized in a pH-dependent manner by a BSA-mediated biomineralization approach, and characterization tests confirmed the successful and proper formation of the desired nanostructures. Bi_2_S_3_@BSA nanoparticles were incorporated into alginate polymer and used as inks in 3D printing. 3D printing allowed Bi_2_S_3_@BSA nanoparticles to spread homogeneously in the alginate scaffold, which has high biocompatibility, biodegradability, and minimal chemical interaction with Bi_2_S_3_@BSA. The application of X-ray alone, which is the standard clinical practice, resulted in modest ROS levels, whereas implantable scaffolds loaded with Bi_2_S_3_@BSA nanoparticles produced a greatly enhanced amount of highly cytotoxic ROS under single-dose X-ray irradiation in vitro. X-ray alone was also ineffective in inhibiting breast cancer cells, killing only ∼20%, whereas the optimized scaffold approximately tripled this effect, killing more than 60% in vitro. To further evaluate the practical applicability, a murine model was employed, in which scaffolds were implanted beneath the tumor tissue of mice and single-dose X-ray irradiation with a dose of 4 Gy was administered. Mice tumor volumes were monitored over time, and scaffolds demonstrated high antitumor efficacy than X-ray alone. The major organs of mice were investigated by histopathology for biocompatibility concerns, and no observable toxicity or adverse effects were reported. Compared to the control groups, tumor tissue exhibited a substantial decrease in tumor cell nucleus size and cytoplasmic structures. Implantable scaffolds may become a viable option for enhanced radiotherapy in clinical applications, particularly to eliminate residual cancer cells remaining in the tumor area post-surgery and to prevent metastasis and may lead to biomaterial-based implant applications in the field of oncology.

## Supporting Information Available

Surgical implantation of the scaffolds (MP4)
DLS analysis of Bi_2_S_3_@BSA nanoparticles (Figure S1); components of the Axo A3 Bioprinting device (Figure S2); 3D printing parameters of the four different hydrogels (Table S1); elemental analyses of scaffolds (Figure S3); in vitro degradation groups (Table S2); and mechanism of ROS detection tests (Figure S4) (PDF)
