# Supplementary material for: Implantable, 3D-Printed Alginate Scaffolds with Bismuth Sulfide Nanoparticles for the Treatment of Local Breast Cancer via Enhanced Radiotherapy
Source: ACS Appl Mater Interfaces. 2024 Mar 20;16(13):15718–29. doi: 10.1021/acsami.3c17024 (PMC10995896; doi:10.1021/acsami.3c17024)
Supplement: Supplementary file 2 — am3c17024_si_002.pdf [file am3c17024_si_002.pdf]

Supporting Information

Implantable, 3D-Printed Alginate Scaffolds with Bismuth Sulfide Nanoparticles for the Treatment of Local Breast Cancer via Enhanced Radiotherapy

Busra Colak[1,2], Yavuz Nuri Ertas[1,2,3*]

[1] ERNAM─Nanotechnology Research and Application Center, Erciyes University, Kayseri 38039, Türkiye

[2] Department of Biomedical Engineering, Erciyes University, Kayseri 38039, Türkiye

[3] UNAM─Institute of Materials Science and Nanotechnology, Bilkent University, Ankara 06800, Türkiye

*Corresponding Author:

Yavuz Nuri Ertas, Ph.D.

ERNAM—Nanotechnology Research and Application Center, Erciyes University, Kayseri 38039, Türkiye

Email: yavuznuri@gmail.com

Web: www.ertaslab.com

Supporting Figures

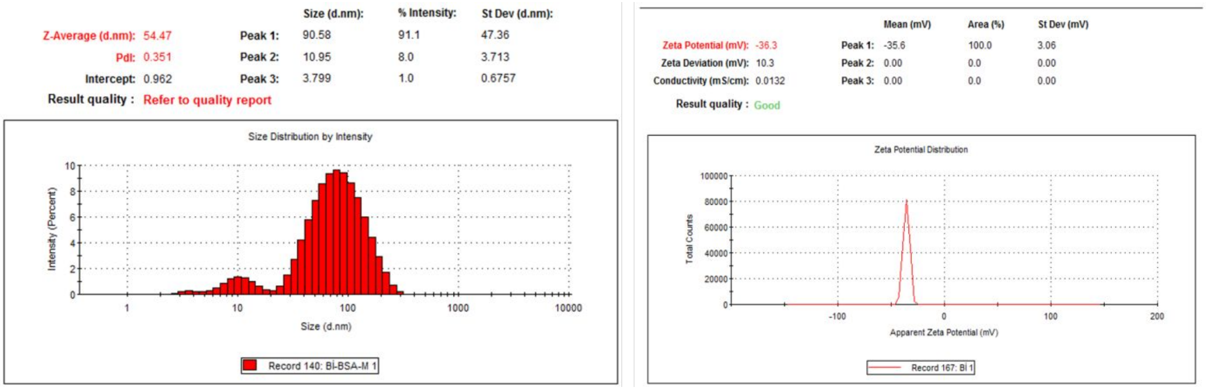

Figure S1. DLS analysis of Bi_2S_3@BSA nanoparticles.

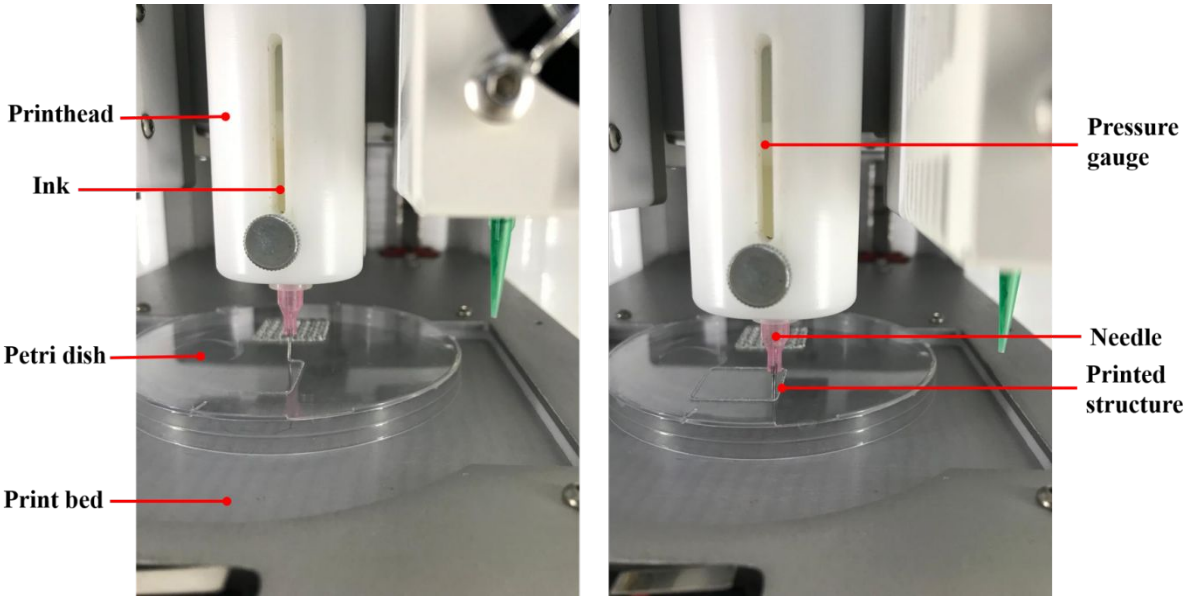

Figure S2. Components of the Axo A3 Bioprinting device.

Table S1. 3D printing parameters of the four different hydrogels.

Conditions	Alginate (Alg)	Alg-Bi_2S_3@BSA (0.25%)	Alg-Bi_2S_3@BSA (0.5%)	Alg-Bi_2S_3@BSA (1%)
**Needle size (G)**	25 G	25 G	25 G	25 G
**Pressure (kPa)**	4.1	5.1	5.8	6.1
**Speed (mm/s)**	100	100	100	100
**Density**	40%	40%	40%	40%
**Print-head temperature (°C)**	-	-	-	-
**Print-bed temperature (°C)**	4	4	4	4

a

Element	Weight %	Atomic %	Net Int.	Error %	Kratio	Z	A	F
C K	13.75	17.69	172.90	12.94	0.0646	1.0431	0.4500	1.0000
N K	0.02	0.03	0.30	99.99	0.0001	1.0186	0.3057	1.0000
O K	85.12	82.20	2976.10	6.76	0.4480	0.9974	0.5277	1.0000
BiM	1.11	0.08	26.50	7.32	0.0088	0.5851	1.3634	0.9959

b

Element	Weight %	Atomic %	Net Int.	Error %	Kratio	Z	A	F
C K	23.32	29.68	65.80	7.30	0.1185	1.0462	0.4857	1.0000
N K	0.00	0.00	0.00	99.99	0.0000	1.0219	0.2074	1.0000
O K	73.35	70.08	370.40	7.59	0.2690	1.0008	0.3665	1.0000
BiM	3.33	0.24	16.60	4.87	0.0267	0.5878	1.3675	0.9964

c

Element	Weight %	Atomic %	Net Int.	Error %	Kratio	Z	A	F
C K	23.98	30.50	65.30	7.28	0.1226	1.0464	0.4884	1.0000
N K	0.00	0.00	0.00	99.99	0.0000	1.0221	0.2028	1.0000
O K	72.53	69.25	344.00	7.68	0.2603	1.0010	0.3584	1.0000
BiM	3.48	0.25	16.70	4.88	0.0279	0.5880	1.3678	0.9965

Figure S3. The data of main elements (C, N, O and Bi) amount in the scaffolds. (a) Alg-Bi_2S_3@BSA (0.25%), (b) Alg-Bi_2S_3@BSA (0.5%), (c) Alg-Bi_2S_3@BSA (1%).

Table S2. *In vitro* degradation groups.

Scaffold Type	Medium	pH	Temperature
**Alginate (Alg)**	PBS (1 ml)	7.4	37 °C
**Alg-Bi_2S_3@BSA (0.25%)**	PBS (1 ml)	7.4	37 °C
**Alg-Bi_2S_3@BSA (0.5%)**	PBS (1 ml)	7.4	37 °C
**Alg-Bi_2S_3@BSA (1%)**	PBS (1 ml)	7.4	37 °C

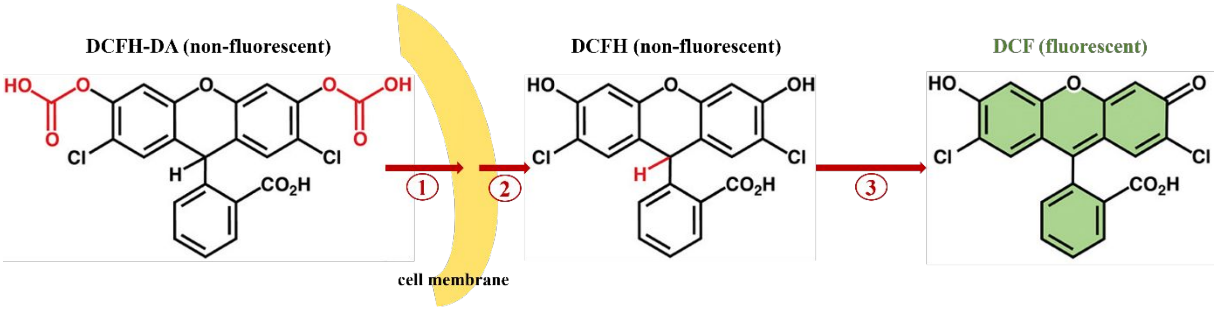

Figure S4. DCFH-DA is taken up by cells **(1)** where cellular esterase cleaves off the acetyl groups, resulting in DCFH **(2)**. Oxidation of DCFH by ROS (such as $^1O_2$, H_2O_2, OH•) converts the molecule to DCF **(3)**, which emits green fluorescence at an excitation wavelength of 485 nm and an emission wavelength of 530 nm.
